# Oral health conditions in an Albanian adolescent population: an epidemiological study

**DOI:** 10.1186/s12903-015-0050-6

**Published:** 2015-06-14

**Authors:** Giuseppina Laganà, Yllka Abazi, Evisi Beshiri Nastasi, Françesca Vinjolli, Francesco Fabi, Maurizio Divizia, Paola Cozza

**Affiliations:** Department of Orthodontics, University of Tor Vergata, Rome, Italy; Private practice, Tirana, Albania; Private practice, Rome, Italy; Department of Experimental Medicine and Surgery, University of Tor Vergata, Rome, Italy; Department of Orthodontics, University of Tor Vergata, Rome, Italy

**Keywords:** IOTN, DMFT, OHI, CPI, PI, Albanian adolescent population, Oral health

## Abstract

**Background:**

The aim of this study was to determine the oral health conditions of an adolescent population of Tirana.

**Methods:**

A cross-sectional epidemiological study was carried out in a sample (n = 1885), aged 16-19, mean age 17.4 (SD = 1.0), attending public schools in Tirana and province; 1200 adolescents were included into the analysis. A clinical observation without radiographs was conducted in the medical room of the schools during the 2012-2013 school year.

**Results:**

Very severe and severe orthodontic treatment need, grade 5 and 4 of IOTN (Index of Orthodontic Treatment Need), were found in 17.0 % of the sample. DMFT (Decayed, Missing and Filled Teeth) was 4.9, whereas OHI (Oral Hygiene Index) was documented in the highest number of subjects (n = 384), 32 % of the total sample possessed “good” grade of oral hygiene. CPI (Community Periodontal Index) was observed at score 0 (healthy gingival condition) in most of the subjects (53.1 %), score 1 (gingival bleeding) in 33.4 % of the total sample. PI (Plaque Index) results showed 43.9 % of the sample (527 subjects) with score 0.

**Conclusions:**

The study findings highlight the need for preventive care programs to improve oral health conditions as well as to reduce oral pathology risk factors in Albania.

## Background

Oral health problems still remain in many communities around the world, particularly among underprivileged groups in developed and developing countries. The significant role of socio-behavioural and environmental factors in oral disease and health is demonstrated in a large number of epidemiological surveys. The global burden of oral conditions increased from 1990 to 2010, collectively affecting 3.9 billion people [1]. Dental caries are still a major oral health problem in most industrialized countries, affecting 60 ± 90 % of schoolchildren and the vast majority of adults [2].

Treatment need indices have been used to plan for the provision of orthodontic treatment in countries in which dental health services are subsidized by the government as part of the national health service or national health insurance system, as is the case in Denmark, Finland, Great Britain, Netherlands, Norway, and Sweden. Because of this connection with public health programs, the use of indices has been limited in countries where publicly funded dental health services are not generally available. However, treatment need indices are also important tools for recording the prevalence and severity of malocclusions in epidemiological studies.

IOTN is used to determine the need or priority for orthodontic treatment in epidemiological surveys. In more recent studies, the IOTN index in relation to different ages has been described in many countries: Spain [3], Brazil [4], United States [5], Iran [6], Malaysia [7] and Albania [8, 9].

The DMFT Index measures the lifetime experience of dental caries in permanent dentition. In many developing countries, access to oral health services is limited and teeth are often left untreated or are extracted because of pain or discomfort. Throughout the world, the loss of teeth is still seen by many people as a natural consequence of aging but it is very important to recognize and lower this index among young people [2].

World Health Organization (WHO) CPI codes are also recorded as an assessment of periodontitis. CPI codes of 0–4 were given: healthy cases and absence of gingival bleeding (score 0), gingival bleeding (score 1), presence of supra or subgingival calculus (score 2), probing pocket depth of 4–5 mm (score 3) or probing pocket ≥6 mm (score 4), respectively. In this WHO classification of periodontitis, it has been determined that the highest CPI code should be selectively applied for each patient by determining all codes in six individual blocks in each periodontal patient [2].

The OHI by Greene [10] is composed of the combined Debris Index and Calculus index, each of these indices is in turn based on 12 numerical determinations representing the amount of debris or calculus found on the buccal and lingual surfaces of each of three segments of each dental arch: the segment distal to the right cuspid, the segment distal to the left cuspid, the segment mesial to the right and left first bicuspids.

The PI system by Sillness defined four levels of quantity and quality of soft deposits: score 0 no plaque, score 1 a film of plaque adhering to the free gingival margin and adjacent areas of the tooth, score 2 moderate accumulation of soft deposits within the gingival pocket or on the tooth and gingival margin which can be seen with the naked eye, score 3 abundance of soft matter within the gingival pocket and/or on the tooth and gingival margin.

Very few epidemiological studies are found which describe oral health conditions and the need of orthodontic treatment in Albania [8, 9, 11]. Laganà found a DMTF value of 2.3 in 7 to 15 year-old students; Hysi found a value of 3.8 in a younger group (12 years old) [12].

The aim of the present study was to determine the orthodontic treatment need using IOTN (Index of Orthodontic Treatment Need) and oral conditions using Decayed Missing Filled Teeth (DMFT), Community Periodontal Index (CPI), Oral Hygiene Index (OHI), Plaque Index (PI), in an adolescent population of Tirana.

## Methods

### Study population

The study target population consisted of 1885 subjects, between 16 and 19 years of age, mean age 17.4 (SD = 1.0), attending public schools in the urban and suburban areas of Tirana (Albania). The thirteen schools examined, eight in the town and five in the surrounding areas of Tirana, were chosen by the Statistical Department of Teaching Direction of Tirana, using a stratified selection technique, in order to represent the right distribution of socio-economical conditions during the 2012-2013 school year. Classes within schools were sampled systematically and all students attending the sampled classes were examined.

### Final sample

Sample size was calculated assuming a 50 percent prevalence ratio for any characteristics. That means an a priori unknown knowledge about the characteristics. The confidence interval for the estimation of the prevalence ratio was assumed as 95 percent.

This assumption led to the highest sample size with precision. One thousand two hundred students, 601 males and 599 females, were randomly selected according to a cluster sampling design. Selection criteria for examinations were: the presence of cuspids and first molars in permanent dentition and no history of orthodontic treatment. Exclusion criteria for this study were: subjects with craniofacial anomalies (syndromes), non-Albanese citizens and students with past or present history of orthodontic treatment.

### Clinical examination

The intraoral examination and orthodontic evaluation were carried out by four examiners. Before undertaking clinical examinations in the schools, the examiners took part in a course on methods of clinical research and orthodontic diagnosis. A pilot study on 50 students was carried out to ensure the accuracy of diagnosis and to standardize the procedures; no statistically significant differences were found (P > 0.05) between the four examiners (Chi-square test of Pearson). The students of each class were randomly assigned and screened by the four examiners. A research assistant observed the examiners throughout the oral examination procedures.

The students were examined in the medical room of the schools on a chair placed very close to the windows in order to use the natural light entering the room. The examination lasted 30 min per subject and the oral and occlusal conditions were assessed by using: latex gloves, plane mouth mirrors, calipers, metallic periodontal probes (CPI probe), in accordance with the WHO guidelines [13]. For each subject, a registration chart was designed which comprised an anamnestic questionnaire and clinical examination measurements, without radiographs, using treatment need indexes such as IOTN, DMFT, OHI, CPI, PI.

### Statistical method

Data were collected by Microsoft Excel 2007 and elaborated by the Statistical Package for the Social Sciences Windows, version 15.0 (SPSS, Chicago, Illinois, USA). Descriptive statistics were calculated for IOTN grades, DMFT, CPI, OHI and PI in order to evaluate the studied sample. Qualitative data were analyzed using the Chi-square test of Pearson to determine if the distributions between age, gender and other variables were statistically different. The P value for statistical significance was set at 0.05, so any value less than P < 0.05 was interpreted as statistically significant.

### Ethics

This research was approved by the Ethics Committee of the Catholic University “Our Lady of Good Counsel” of Tirana.

A written consent was obtained from the students and their parents before starting clinical examinations.

## Results

A total of 1885 students were examined, 685 of them (36.3 %) were not included in the study for diverse reasons such as lack of a first molar/canine, present or past orthodontic treatment or no written consent. The final studied sample was composed of 1200 subjects, 601 males (50.1 %) and 599 females (49.9 %). Table 1 reports IOTN evaluation according to the age (16-19 years) and gender (males-females). An objective treatment need (grades 5 and 4) was recorded respectively in 203 subjects, 17.0 % of the total sample. If we consider IOTN evaluation, according to the age (16-19 years), the highest number of subjects with grade 1 was present at eighteen years old, whereas the lowest number of subjects, just one, is recorded at sixteen years. The eighteen and nineteen year-old group presented grade 5 of IOTN (very great treatment need). The association with age was statistically significant (Table 1).

**Table 1:** Distribution of IOTN according to age and gender

IOTN (DHC)	16 years		17 years		18 years		19 years		Males		Females		Total	
	Nr.		Nr.		Nr.		Nr.		Nr.		Nr.		Nr.	
	(%)		(%)		(%)		(%)		(%)		(%)		(%)	
Grade 1	102	(8.5)	158	(13.1)	163	(13.6)	59	(4.9)	226	(18.8)	256	(21.3)	482	(40.0)
Grade 2	71	(5.9)	122	(10.1)	134	(11.2)	56	(4.7)	192	(16.0)	191	(15.9)	383	(32.0)
Grade 3	34	(2.8)	51	(4.2)	27	(2.2)	20	(1.5)	71	(5.9)	61	(5.1)	132	(11.0)
Grade 4	47	(3.9)	67	(5.6)	47	(3.9)	31	(2.6)	109	(9.1)	83	(6.9)	192	(16.2)
Grade 5	1	(0.1)	8	(0.7)	1	(0.1)	1	(0.1)	3	(0.2)	8	(0.7)	11	(0.8)
Total	255	(21.2)	406	(32.7)	372	(31.0)	167	(13.8)	601	(50.0)	599	(49.9)	1200	(100)

AGE - *χ* ^2^: 25.2; D.F.: 12; p-value: 0.014

The total DMFT value in the studied subjects was 4.9, 4.8 in females and 4.9 in males (Table 2); the highest value can be observed in 19 year-old subjects (DMFT = 5.3), the lowest in 16 year-old subjects (DMFT = 4.4). As expected, the highest value of DMFT is related with a “Not Good” grade of OHI.

**Table 2:** Prevalence of DMFT according to age and gender

Sample		Decayed		Missed		Filled		D + M + F		DMFT
		Nr.		Nr.		Nr.		Nr.		
		(%)		(%)		(%)		(%)		
16 years	255	545	(9.3)	16	(0.3)	566	(9.7)	1127	(19.3)	4.4
17 years	406	770	(13.2)	12	(0.2)	1112	(19.0)	1894	(32.4)	4.7
18 years	372	679	(11.6)	21	(0.4)	1236	(21.2)	1936	(33.2)	5.2
19 years	167	305	(5.2)	15	(0.2)	562	(9.6)	882	(15.1)	5.3
Males	601	1187	(20.3)	31	(0.5)	1734	(29.7)	2952	(50.6)	4.9
Females	599	1112	(19.0)	33	(0.6)	1742	(29.8)	2887	(49.4)	4.8
Total	1200	2299	(39.4)	64	(1.1)	3476	(59.5)	5839	(100)	4.9

The prevalence of CPI according to age and gender is described in Table 3. Females showed better periodontal conditions than males and this difference was statistically significant.

**Table 3:** Prevalence of CPI according to age and gender

CPI	16 years		17 years		18 years		19 years		Males		Females		Total	
	Nr.		Nr.		Nr.		Nr.		Nr.		Nr		Nr.	
	(%)		(%)		(%)		(%)		(%)		(%)		(%)	
0	128	(10.7)	214	(17.8)	206	(17.2)	89	(7.4)	295	(24.6)	342	(28.5)	637	(53.1)
1	88	(7.3)	136	(11.3)	115	(9.6)	61	(5.1)	201	(16.7)	199	(16.6)	400	(33.4)
2	36	(3.0)	55	(4.6)	49	(4.1)	17	(1.4)	104	(8.7)	53	(4.4)	157	(13.1)
3	3	(0.2)	0	(0.0)	2	(0.2)	0	(0.0)	0	(0.0)	5	(0.4)	5	(0.4)
4	0	(0.0)	1	(0.1)	0	(0.0)	0	(0.0)	1	(0.1)	0	(0.0)	1	(0.1)
Total	255	(21.2)	406	(32.8)	372	(31.1)	167	(13.9)	601	(50.1)	599	(49.9)	1200	(100)

GENDER - *χ* ^2^: 26.0; D.F.: 4; p-value: 0.000

OHI results according to age and gender are provided in Table 4. Most of the sample (384 subjects, 32.1 % of the studied sample) showed a “Good” score. Females are better than males in maintaining oral hygiene: 206 females presented a “Good” score and 165 “Very Good” compared to 178 and 108 males, respectively.

**Table 4:** Prevalence of OHI and PI according to age and gender

	16 years		17 years		18 years		19 years		Males		Females		Total	
	Nr.		Nr.		Nr.		Nr.		Nr.		Nr		Nr.	
	(%)		(%)		(%)		(%)		(%)		(%)		(%)	
ORAL HYGIENE INDEX (OHI)														
Very good	51	(4.2)	93	(7.7)	92	(7.8)	37	(3.1)	108	(9.0)	165	(13.7)	273	(22.7)
Good	83	(6.9)	138	(11.5)	113	(9.4)	50	(4.1)	178	(14.8)	206	(17.2)	384	(32.1)
Sufficient	69	(5.7)	95	(7.9)	91	(7.6)	50	(4.2)	161	(13.4)	144	(12.0)	305	(25.4)
Not good	52	(4.3)	80	(6.7)	76	(6.3)	30	(2.5)	154	(12.8)	84	(7.0)	238	(19.8)
Total	255	(21.1)	406	(33.8)	372	(31)	167	(13.9)	601	(50.2)	599	(49.9)	1200	(100)
	PLAQUE INDEX (PI)													
Score 0	106	(8.8)	189	(15.7)	158	(13.2)	74	(6.2)	223	(18.6)	304	(25.3)	527	(43.9)
Score 1	60	(5.0)	80	(6.7)	91	(7.6)	33	(2.7)	126	(10.5)	138	(11.5)	264	(22.0)
Score 2	66	(5.5)	92	(7.7)	81	(6.7)	44	(3.7)	165	(13.7)	118	(9.8)	283	(23.6)
Score 3	23	(1.9)	45	(3.7)	42	(3.5)	16	(1.3)	87	(7.2)	39	(3.2)	126	(10.5)
Total	255	(21.2)	406	(33.8)	372	(31.0)	167	(13.9)	601	(50.0)	599	(49.8)	1200	(100)

Table 4 also describes PI according to age and gender: 527 subjects, 43.9 % of the total sample, presented “Score 0”. Females performed better than males in this evaluation.

Significant correlations were observed between the results of IOTN and the other indices used in the clinical examination of our sample. By using the Chi-square test, IOTN was found to be statistically significant showing the dependency with the following variables: IOTN grade 1 and OHI (*χ*^2^ = 611.5, P value < 0.0001), IOTN grade 1 and CPI (*χ*^2^ = 605.1, P value < 0.0001), IOTN grade 2 and CPI (*χ*^2^ = 1214.9, P value < 0.0001), IOTN grade 3 and OHI (*χ*^2^ = 1222.7, P value < 0.0001), IOTN grade 3 and CPI (*χ*^2^ = 1219.6, P value < 0.0001), IOTN grade 4 and OHI (*χ*^2^ = 1253.4, P value < 0.0001), IOTN grade 4 and CPI (*χ*^2^ = 1260.5, P value < 0.0001), IOTN grade 5 and OHI (*χ*^2^ = 1261.3, P value < 0.0001), IOTN grade 5 and CPI (*χ*^2^ = 1261.3, P value < 0.0001).

## Discussion

This study provides information about oral health conditions of 16 to 19-year-old students, highlighting the low level of prevention and poor oral health in Albanian adolescents.

The percentage of subjects in need of orthodontic treatment in our study (17.0 %) was comparable with the few previous investigations that had used IOTN. Our data were lower than in studies of European [3] and Non European countries [6, 7, 14, 15]. The subjective nature of aesthetic OTNI (Orthodontic Treatment Need Indices) and the minor contributory role of malocclusion in psychosocial health or quality of life undermine the use of aesthetic indices to assess the likely social and psychological implications of malocclusion. Consequently, using aesthetic OTNI as a method to quantify malocclusion remains open to debate [16].

DMFT in our sample (4.9) is different from other general results from Western European Regions (1.9) but similar to the values reported in other Eastern European Regions such as Slovakia, Serbia and Macedonia [17]. A similar DMFT value found in Albania was recorded by Bolin [18] in adolescent detainees in Texas where the “community prisoners” had a low utilization of preventive or therapeutic dental services. In previous studies concerning the prevalence of caries in Albania, Hysi [8] in 12 year-old students reported a value of 3.8 whereas Laganà [12] reported that in 7-15 year old students, the value was only 2.3. On the other hand, Thelen [19] in a similar sample (16-19 year old students) evaluated the DMTF value to be 4.6. In Albania during the period 1990-2007, DMFT increased from 2.8 to 3.8. This tendency could be explained by better clinical examinations of dental conditions, which were absent or incomplete in the past.

As expected, the highest value of DMFT (5.3 in 19 year old group) is correlated to a “Sufficient” grade of OHI. The level of oral hygiene resulted “Good” in most of the subjects and, as predicted, the group with the “Very Good” score in OHI presented the lowest value in the DMFT index.

Contrary to all expectations, the CPI results indicated healthy periodontal conditions, showing more than half of the examined subjects (53.1 %) with normal conditions (grade 0). These results are lower if compared with results in other countries such as Greece [20], Germany [21], Italy [22], Hungary [17].

Plaque Index results showed a high percentage of subjects with score 0, which indicates the absence of plaque. Significant differences are observed between gender and age: females and students aged eighteen are better in maintaining good oral hygiene. Other studies indicate an improving tendency of oral health-behaviour/tooth brushing after the initial years of adolescence [23].

As in other studies [24], the association between IOTN and other variables was observed; these findings suggest that the relationship between caries and malocclusions should be assessed in a wider context, such as parental education, mother’s employment status and background factors.

Some limitations of the present study should be considered when interpreting the results including the reliability of the clinical measurements of malocclusion, such as the Index of Orthodontic Treatment Need (IOTN), and Angle’s classification of malocclusion. Visible proximal caries were included by using the clinical criteria to diagnose proximal caries, but bitewing radiographs were not taken to determine exact proximal caries. According to the WHO Oral Health Surveys Basic Methods 1997, radiographs are not required and dental caries are measured using dentition status and treatment needs [13]. The omission of radiographic diagnosis in epidemiological studies of dental caries in young adolescent populations with high caries experience (or where it is suspected to be high) is likely to lead to considerable underestimation of caries occurrence, particularly with respect to the severity of caries (as represented by mean DMF scores) [25]. Moreover, the superior OHI, CPI and PI results compared to those of the DMFT can be explained: the students knew that screenings were to be scheduled in their school and this could have modified their usual oral hygiene practices.

In line with goals of WHO, recommending to integrate oral health promotion and educational programs into school activities, the authors offer the following proposals for Albania:

1. Integrate educational oral health care programs in all public and private schools;
2. Prepare and distribute simple guidelines for oral health care as well as information about malocclusions to parents and teachers;
3. Initiate annual dental check-ups for children beginning at age six;
4. Send specialists to primary schools for overall clinical evaluations;
5. Create and provide training about orthodontic early treatment to sensitize paediatrician and general family practitioners in recognizing malocclusion development risk factors;
6. Assess target populations at higher risk for oral diseases through the use of database systems and epidemiological studies in different areas of the country.

These recommendations may prove challenging for the Dental Public Health Service in Albania due to social, economic and cultural factors. But access to quality dental care is paramount to meeting the health needs of the population.

## Conclusions

The results of this study describes low levels of oral health in Albanian adolescents. These poor oral health conditions can be improved with early detection and preventive care. In fact, WHO recommends integrating oral health promotion and educational programs into school activities with the aim of successfully reaching the goal of minimizing the impact of oral and craniofacial diseases on general health and psycho-social well-being by 2020 [19]. The high degree of orthodontic treatment need in Albania should also be addressed. Our indications may hopefully improve the strategies for oral disease prevention and health promotion in the near future.

### Availability of supporting data

Clinical data of studied subjects are available in Labarchives as “Clinical database of adolescents of Tirana -Laganà *et al.*” http://dx.doi.org/10.6070/H41J97Q0.
